# Trends and sex‐specific incidence of upper urinary tract cancer in Taiwan: A birth cohort study

**DOI:** 10.1002/cam4.6084

**Published:** 2023-07-01

**Authors:** Yu‐Hsuan Chang, Wan‐Lun Hsu, Yu‐Kwang Lee, Chun‐Ju Chiang, Ya‐Wen Yang, San‐Lin You, Yong‐Chen Chen, Tai‐Shuan Lai

**Affiliations:** ^1^ School of Medicine Fu‐Jen Catholic University New Taipei City Taiwan; ^2^ Data Science Center, College of Medicine Fu‐Jen Catholic University New Taipei City Taiwan; ^3^ Master Program of Big Data Analysis in Biomedicine, College of Medicine Fu‐Jen Catholic University New Taipei City Taiwan; ^4^ Department of Surgery, Division of General Surgery National Taiwan University Hospital Taipei Taiwan; ^5^ Graduate Institute of Epidemiology and Preventive Medicine, College of Public Health National Taiwan University Taipei Taiwan; ^6^ Department of Internal Medicine National Taiwan University Hospital Taipei Taiwan; ^7^ Department of Internal Medicine, College of Medicine National Taiwan University Taipei Taiwan

**Keywords:** age period cohort, incidence, sex, upper tract urothelial cancer

## Abstract

**Background:**

Taiwan has one of the highest incidences of upper tract urothelial cancer (UTUC) worldwide, especially in women; however, no nationwide, long‐term follow‐up study has evaluated this.

**Methods:**

We investigated the incidence of UTUC in Taiwan using data from the national population‐based Taiwan Cancer Registry database (1985–2019). We divided the birth cohort into nine 5‐year age groups and calculated the age‐specific incidence for these groups according to the corresponding birth years.

**Results:**

The average annual percent change in the incidence of renal pelvis cancer from 1985 to 2019 showed sex‐specific differences, with 3.5% and 5.3% increases in the incidences in men and women, respectively. The age‐specific incidence rate for renal pelvis cancer among women showed a gradual increase in the group with older women as well as an increase over time in each age group. The results of a birth cohort analysis revealed that younger cohorts had higher incidence rates of renal pelvis cancer than older cohorts did.

**Conclusion:**

We demonstrated that the incidence of UTUC is unusually high among older Taiwanese women and that younger cohorts have a high risk of UTUC than older cohorts.

## 1 INTRODUCTION

Urothelial cancer is the most common malignant tumor of the urinary tract and one of the most prevalent cancers worldwide. Although most urothelial cancers occur in the bladder, approximately 5% of all cases occur in the upper urinary tract organs (e.g., the renal pelvis and ureter).^1^ Urothelial carcinomas of the upper urinary tract have been regarded as relatively rare. However, compared with those in other countries, the incidence of upper tract urothelial cancer (UTUC) in Taiwan is abnormally high, especially in women.^2^, ^3^, ^4^, ^5^, ^6^ The annual incidence of UTUC is 1–2 cases per 100,000 people worldwide, but it is 3.14–3.41 in Taiwan.^7^, ^8^, ^9^ Previous studies have shown that there are sex‐specific differences in UTUC, with the prevalence of UTUC being approximately twice as high in males than in females in the United States and Western European.^7^, ^10^, ^11^ In contrast, a 2001–2010 Taiwanese study showed that the annual incidence of UTUC in women was significantly higher than that in men (female to male ratio: 2.08–3.25).^9^ Relevant studies conducted in recent years have determined that the carcinogenic mechanisms of UTUC are related to smoking, exposure to aristolochic acid, genetic predisposition, and environmental factors.^12^, ^13^ A previous study showed a pooled odds ratio (OR) of 5.97 (95% confidence interval [CI] = 2.78–12.84) for the association between aristolochic acid and UTUC.^14^ Another study reported that end‐stage kidney disease (ESKD) increased the risk of developing UTUC and showed an adjusted hazard ratio of 3.38 (95% CI = 2.38–4.81) for the association between ESKD and UTUC.^15^

Previous studies in Taiwan have evaluated changes and trends in the incidence of UTUC^2^, ^9^ but have not analyzed the higher incidence of UTUC in Taiwanese women than in women from other countries as well as the peculiar age‐specific trends in the incidence of UTUC in Taiwanese women. To understand the reasons and peculiar trends observed regarding UTUC incidence, we evaluated long‐term incidence trends for UTUC and investigated the potential reasons underlying the observed trends in this study. We specifically discussed the trends in the incidence of UTUC in Taiwanese women.

## 2 MATERIALS AND METHODS

### 2.1 Data sources

We conducted this study using UTUC‐related data from the national population‐based Taiwan Cancer Registry (TCR) database (1985–2019). Since 1979, the TCR has worked with hospitals with capacities of more than 50 beds to establish a cancer registration system.^16^ Patient information in the TCR comprises demographic and diagnostic data. The completion rate of the TCR database reached 98.35% in 2019. Regarding cases of UTUC confirmed by cytology or histopathology, the confirmation rate was reported to be 96.92%.^17^ The patients with cancer selected in the current study were classified according to the International Classification of Diseases for Oncology, third edition,^18^ and were divided into the following categories: renal pelvis (C65) and other urinary organs (C66 and C68). From 1985 to 2019, the total number of cases of renal pelvis cancer, and cancers of other urinary organs in Taiwan was 17,267 and 16,275, respectively.

### 2.2 Statistical analyses

We calculated the age‐specific and age‐adjusted incidence rates for renal pelvis cancer and cancers of other urinary organs in Taiwan. The incidence data were divided into 18 5‐year age groups. We divided the cohort into eight groups as per the birth years: 1935–1939, 1940–1944, 1945–1949, 1950–1954, 1954–1959, 1960–1964, 1965–1969, and 1970–1974, and calculated the age‐specific incidence rates for these birth‐year cohorts limited to nine 5‐year age groups (40–44, 45–49, 50–54, 55–59, 60–64, 65–69, 70–74, 75–79, and 80–84 years). Age‐adjusted incidence rates were calculated according to the 2000 World Health Organization standard population data.^19^ Incidence rates are presented as a rate per 100,000 person‐years. Moreover, we calculated the annual percent change (APC) and average APC (AAPC) to describe the linear trends over time observed in the present study. The 95% CI of AAPC, excluding 0, was considered statistically significant (*p* < 0.05). In this study, the Joinpoint Trend Analysis Software developed by the National Institutes of Health was used to calculate the APC (v.4.9.0.1; https://surveillance.cancer.gov/joinpoint/). All data analyses were performed using the SAS version 9.4 software (SAS Institute).

There was no patient or public involvement in this study. This study used open data available from the Health and Welfare Data Science Center, Taiwan; due to legal restrictions imposed by the government of Taiwan in relation to the “Personal Information Protection Act,” the data are anonymized, personal information cannot be made publicly available, and researchers have no access to individual cases. The study protocol was approved by the institutional review board (IRB) of Fu‐Jen Catholic University (no. C107099), and this work was conducted in accordance with the principles of the Declaration of Helsinki and its later amendments. The requirement for obtaining patient consent was waived because this study used a registry database with encrypted personal identifiers, and the IRB consequently exempted the study from review.

## 3 RESULTS

**FIGURE 1:**
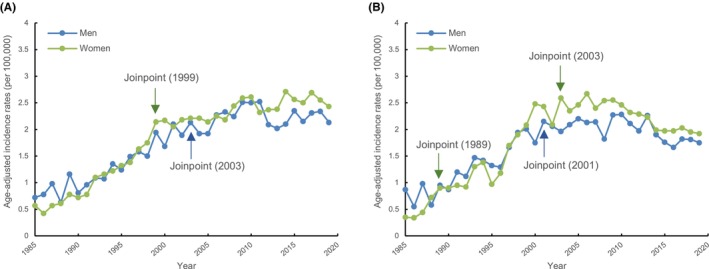
(A) Long‐term trends in age‐adjusted incidence rates for renal pelvis cancer by sex. (B) Long‐term trends in age‐adjusted incidence rates for cancers of other urinary organs by sex.

As shown in Figure 1, long‐term trends in the age‐adjusted incidence of renal pelvis cancer and cancers of other urinary organs revealed that the incidences in women gradually exceeded those in men. According to the APC analysis, the incidence of renal pelvis cancer in women increased rapidly from 1985 to 1999 (APC = 11.3%, 95% CI = 10.1–12.5; Table 1), with a steady trend from 1999 to 2019 (APC = 1.3%, 95% CI = 0.6–1.9). In contrast, the age‐adjusted incidence of renal pelvis cancer increased more slowly in men than women, and the joinpoint for APC occurred in 2003, later than that in women (APC = 6.4%, 95% CI = 5.2–7.6; Table 1). Overall, the AAPC of the incidence of renal pelvis cancer in women from 1985 to 2019 was 5.3%, which was higher than that in men (3.5%; Table 1).

**TABLE 1:** Sex‐specific annual percent change (APC) and average annual percent change (AAPC) for the incidence of urinary tract cancers by sex in Taiwan between 1985 and 2019.

Cancer site	Sex	Joinpoint segment initial year	Joinpoint segment final year	APC (95% confidence interval [CI])	*p*‐Value	AAPC over the whole period	*p*‐Value
Renal pelvis	Men	1985	2003	6.4 (95% CI = 5.2–7.6)	<0.001	3.5 (95% CI = 2.7–4.3)	<0.001
		2003	2019	0.3 (95% CI = −1.0–1.6)	0.623		<0.001
	Women	1985	1999	11.3 (95% CI = 10.1–12.5)	<0.001	5.3 (95% CI = 4.7–5.9)	<0.001
		1999	2019	1.3 (95% CI = 0.6–1.9)	<0.001		<0.001
Other urinary organs	Men	1985	2001	7.6 (95% CI = 6.0–9.3)	<0.001	3.0 (95% CI = 2.0–3.9)	<0.001
		2001	2019	−1.0 (95% CI = −2.3 to 0.3)	0.119		<0.001
	Women	1985	1989	27.5 (95% CI = 14.2–42.5)	<0.001	5.6 (95% CI = 3.9–7.2)	<0.001
		1989	2003	8.9 (95% CI = 6.9–10.9)	<0.001		<0.001
		2003	2019	−2.0 (95% CI = −3.3 to −0.7)	0.004		<0.001

**FIGURE 2:**
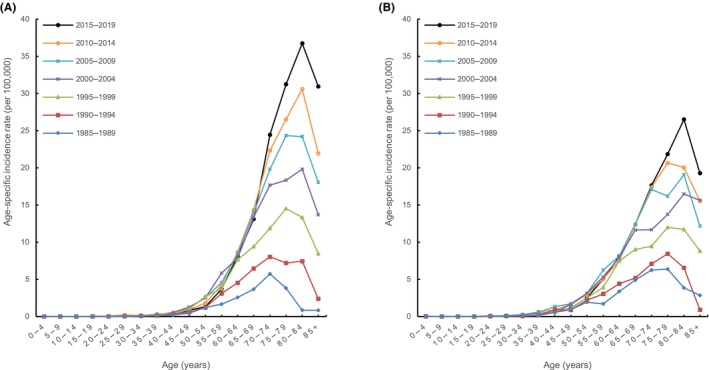
Age‐specific incidence rates for renal pelvis cancer (A) in women and (B) in men.

We then compared the age‐specific incidence rates of renal pelvis cancer among women from 1985 to 2019 and found that the incidence rates in the older age groups gradually increased (Figure 2A). On comparing the age‐specific incidence rates of renal pelvis cancer in women in the different periods evaluated, we found that the incidence rate for each age group increased with time, especially in those older than 70 years. The increase in the incidence rate with age was more evident over time. Additional analyses demonstrated that the age required to achieve the same age‐specific incidence rate for renal pelvis cancer in women gradually decreased over time. From 2000 to 2004, the age‐specific incidence rate for women aged 80–84 years was 19.82 per 100,000 persons; however, from 2005 to 2009, women aged 70–74 years nearly reached this rate (19.8 per 100,000 persons), representing a 10‐year difference in reaching the same rate. This trend in age‐specific incidence rates by period regarding renal pelvis cancer was irregular and less evident in men than in women (Figure 2B). The increasing trend in terms of the incidence rate in older age groups over time was not as evident as that in the female population. For cancers of other urinary organs in men and women, evaluations of the age‐specific incidence rates by period did not show trends similar to those seen in women with renal pelvis cancer (Figure S1).

**FIGURE 3:**
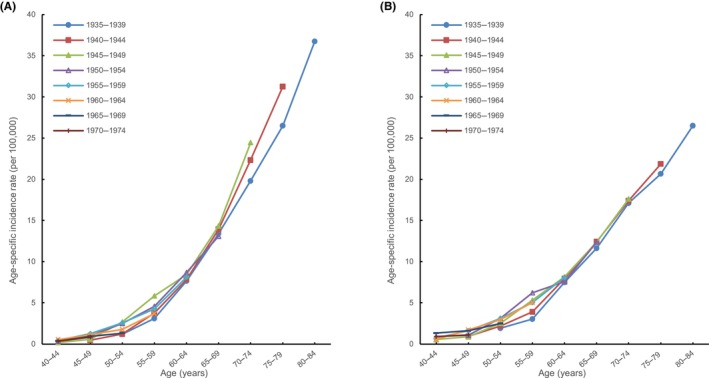
Age‐specific incidence rates by birth‐year cohorts between 1935 and 1974 for renal pelvis cancer (A) in women and (B) in men.

Finally, we analyzed the age‐specific incidence rates for renal pelvis cancer in Taiwan by birth year. Among women of the same age group in the older age groups, the incidence rates for the younger birth‐year cohorts were higher than those for the older birth‐year cohorts. This trend was more noticeable in the groups of women aged over 70 years (Figure 3A). This trend became more evident with increasing age. In men, age‐specific incidence rates for renal pelvis cancer by birth year did not show this trend nor did evaluation of the incidence of cancers of other urinary organs by birth year in both men and women (Figure 3B, Figure S2).

## 4 DISCUSSION

Our study revealed that the incidence of UTUC was high in women, especially for renal pelvis cancer in Taiwanese women from 1985 to 2019. The incidence of renal pelvis cancer in women showed an upward trend from 1985 to 1999, with an APC of 11.3% (95% CI = 10.1–12.5). This upward trend declined significantly to 1.3% from 1999 to 2019. The incidence rate for each age group increased with time, especially in those older than 70 years. The increasing trend in the incidence rate with age was more evident over time. In the birth‐year cohort analysis, the incidence rates for younger birth‐year cohorts were higher than those for older birth‐year cohorts. This trend was more evident in the groups aged over 70 years.

In the Cancer Incidence in Five Continents Volume XI,^20^ which did not include Taiwan, the regions with the highest age‐standardized incidences of renal pelvis cancer in men and women from 2008 to 2012 were 1.6 and 1.2 in the Niigata Prefecture in Japan and in Nebraska in the United States, respectively. The regions with the highest age‐standardized incidence of cancers of the ureter in men and women from 2008 to 2012 were 1.4 and 1.3 in the Hiroshima Prefecture in Japan and in the Northwest Territories in Canada, respectively. In 2012, the standardized incidence rates of renal pelvis cancer and cancers of the ureter were 2.87 and 1.9, respectively, in Taiwan.^21^ This international comparison shows that the incidence of UTUC in Taiwan is unusually higher than those in other countries. A comparative study conducted in Taiwan and Japan showed that patients with UTUC in Taiwan were younger (on average) than those in Japan and included a higher proportion of women.^22^ In our study, we found a progressive increase in the incidence in older women from 1985 to 2019, which increased over time in each older age group.

These results indicate that the risk factors for UTUC in women in Taiwan differ from those in other countries, and Taiwanese women may be more susceptible than Taiwanese men. The European Association of Urology indicated that for UTUC to develop, the average required risk factor exposure time is approximately 7 years, and there may be a latency period of approximately 20 years after cessation of exposure.^23^ Considering the above‐mentioned cancer induction time, our study made a chronological deduction regarding the following relevant risk factors: aristolochic acid exposure, chronic dialysis, aromatic amine exposure, and arsenic exposure.

Aristolochic acid exposure is an important risk factor for UTUC.^24^ Although the pathogenic mechanisms are not fully understood, previous studies have demonstrated that A→T transversions and increased aristolactam–DNA adducts caused by aristolochic acid exposure are associated with the occurrence of UTUC.^25^, ^26^ In one study, individuals exposed to aristolochic acid showed an approximately six‐fold higher risk of developing UTUC than that shown by those not exposed to aristolochic acid.^14^ The use of traditional Chinese medicines (TCMs) containing aristolochic acid has been demonstrated to be highly correlated with the incidence of UTUC.^27^, ^28^, ^29^ A study showed that the prescription frequency of aristolochic acid herbal medicine from 1997 to 2003 was 31.6 per 1000 person‐years for women, which was higher than that for men (26.9 per 1000 person‐years).^30^ Studies regarding the age of individuals using aristolochic acid showed that from 1997 to 2003, the groups with the highest frequency of AA‐CHP use were those aged 35–59 years. Among this age group, the prescription frequency was 20.6 per 1000 person‐years.^30^ Another study in Taiwan found that Taiwanese menopausal women used TCM more often than women of other age groups.^31^ The above‐mentioned study showed that between 1997 and 2003, Taiwanese women in their 50s and 60s may have had greater exposure to aristolochic acid than did those in other age groups, and these women correspond to the birth‐year cohorts between 1940 and 1950. Higher midlife exposure to aristolochic acid among women in the 1940–1950 birth‐year cohort provides a possible explanation for the higher incidence of renal pelvis cancer in this age group in our study.

According to the 2020 U.S. Renal Data System Annual Data Report, Taiwan has the second‐highest incidence of ESKD worldwide.^32^ Previous studies have shown that impaired DNA repair, lowered immune system function, decreased antioxidant capacity, and dialysis‐related complications caused by ESKD may be potential mechanisms mediating malignant transformation.^33^ A population‐based cohort study conducted in Taiwan in 2016 confirmed that chronic kidney disease (CKD) and ESKD increase the risk of UTUC developing and showed that the adjusted hazard ratios for CKD and ESKD regarding UTUC were 1.63 (95% CI = 1.26–2.13) and 3.38 (95% CI = 2.38–4.81), respectively.^15^ A sex‐specific difference in risk was also detected. Women with ESKD have a 9–18‐fold increased risk of developing urothelial cancer, which is higher than the 4–14‐fold increased risk observed in men.^34^ Therefore, the substantial difference in the prevalence of ESKD between men and women in Taiwan may explain the increasing trend in the incidence of UTUC among older women in Taiwan.

Exposure to aromatic amines is considered a risk factor for UTUC,^35^ and occupational exposure may be a potential route. A previous study in Japan showed that occupational exposure to aromatic amines increases the risk of urothelial tumors (OR: 8.302).^36^ In Taiwan, industries and factories were being developed increasingly in the 1970s. According to the Directorate General of Budget, Accounting, and Statistics, the textile and electronics industries were the top two employers of women in Taiwan from 1981 to 1996.^37^ Previous studies have shown that workers in the textile and electronics industries are exposed to higher levels of aromatic amines than the general population.^38^, ^39^, ^40^ Many women born in the 1940s and 1950s entered the workforce during this period; therefore, chronic exposure to aromatic amines at their workplaces may account for the higher incidence of UTUC observed at older ages in this birth‐year cohort than in previous birth‐year cohorts.

Some studies have demonstrated that exposure to arsenic results in the production of reactive oxygen and nitrogen species, which may lead to DNA damage.^41^ Moreover, exposure to arsenic has been highly correlated with the occurrence of UTUC and other cancers.^42^, ^43^ The southwestern coast of Taiwan is an endemic area for “black foot disease,” which is caused by long‐term exposure to (i.e., consumption of) arsenic‐containing groundwater.^44^, ^45^ Statistics show a relatively high incidence of UTUC in this area.^2^ However, there was no significant difference in the overall arsenic exposure between Taiwanese men and women; therefore, arsenic exposure could not fully explain the increasing trends in the incidence of UTUC in older Taiwanese women.

A notable strength of our study is the high confirmation rate within the TCR database. The second major strength is our access to ecological data on risk factor exposures relevant to UTUC during the corresponding induction period. Therefore, the estimated exposure time was in line with the latency period for carcinogenesis, and we were able to perform a chronological deduction regarding the incidence of UTUC. The present study had some limitations. First, the database has limitations in terms of data on cancer registration. If a patient has more than two urothelial cancer types simultaneously, only the earliest cancer type will be registered, which may theoretically lead to an underestimation of the incidence of renal pelvis cancer and cancers of other urinary organs. However, this potential limitation likely had a limited impact on our trend analysis of age‐standardized incidence and birth‐year cohort‐specific evaluation of age‐specific incidence rates. Second, the population data in the TCR database routinely use 5‐year intervals. Therefore, we used 5‐year intervals as the basis for calculating the incidence rates. Third, because the TCR database does not include personal‐level data of patients with cancer, we could not evaluate individualized factors such as diet or lifestyle habits and therefore did not have complete risk‐factor exposure data. Individual differences within a population may lead to an ecological fallacy, and there is no efficient way to adjust for such factors. Hence, we employed an overall trend analysis to explain changes in UTUC incidence trends using a causal time series. Future studies with complete exposure data are required for more comprehensive evaluations. Fourth, we did not compare the incidence trends of other forms of urothelial carcinoma such as bladder cancer in the same population. Future studies are recommended to further analyze and evaluate the incidence of other forms of urothelial carcinoma. Finally, the various risk factors associated with UTUC in Taiwan may be different from those in other countries, and correlations among these risk factors have not been fully determined. Therefore, our results may not be generalizable to other countries, and more research focusing on transnational cooperation is required.

In conclusion, our study revealed that the incidence of UTUC in Taiwan is high, especially in renal pelvis cancer in women. The incidence of renal pelvis cancer in women increased rapidly from 1985 to 1999 and slowed down from 1999 to 2019. The incidence rate for each age group increased with time, especially in those older than 70 years. The incidence rates for the younger birth‐year cohorts were higher than those for the older birth‐year cohorts. Thus, early detection and surveillance should be performed to target vulnerable age and sex groups. Further studies are warranted to explore possible reasons for the peculiar characteristics of UTUC incidence in Taiwan.

## AUTHOR CONTRIBUTIONS

**Yu‐Hsuan Chang:** Conceptualization (equal); writing – original draft (equal); writing – review and editing (equal). **Wan‐Lun Hsu:** Conceptualization (supporting); data curation (supporting); methodology (supporting); writing – review and editing (supporting). **Yu‐Kwang Lee:** Conceptualization (equal); formal analysis (supporting); methodology (supporting); writing – review and editing (supporting). **Chun‐Ju Chiang:** Data curation (lead); formal analysis (lead); writing – review and editing (supporting). **Ya‐Wen Yang:** Conceptualization (equal); data curation (supporting); formal analysis (supporting); resources (supporting). **San‐Lin You:** Conceptualization (supporting); data curation (supporting); formal analysis (supporting); resources (supporting); writing – review and editing (supporting). **Yong‐Chen Chen:** Conceptualization (equal); methodology (lead); project administration (lead); resources (supporting); writing – review and editing (supporting). **Tai‐Shuan Lai:** Conceptualization (lead); funding acquisition (lead); resources (equal); writing – original draft (equal); writing – review and editing (lead).

## FUNDING INFORMATION

This study received no external funding.

## ETHICS STATEMENT

The research protocol for this study was approved by the institutional review board of Fu‐Jen Catholic University (no. C107099), and this work was conducted in accordance with the principles of the Declaration of Helsinki and its later amendments.

## Supporting information


Figure S1.
Figure S2.

## Data Availability

Data are available from the Health and Welfare Data Science Center (HWDC). Due to the legal restrictions imposed by the government of Taiwan in relation to the “Personal Information Protection Act,” data cannot be made publicly available. Requests for data can be sent as formal proposals to https://dep.mohw.gov.tw/DOS/lp‐25.
