# Supplementary material for: Trends and sex‐specific incidence of upper urinary tract cancer in Taiwan: A birth cohort study
Source: Cancer Med. 2023 Jul 1;12(14):15350–7. doi: 10.1002/cam4.6084 (PMC10417072; doi:10.1002/cam4.6084)
Supplement: Supplementary file 1 — Figure S1. Figure S2. [file CAM4-12-15350-s001.pdf]

Supplemental material

(A) (B)

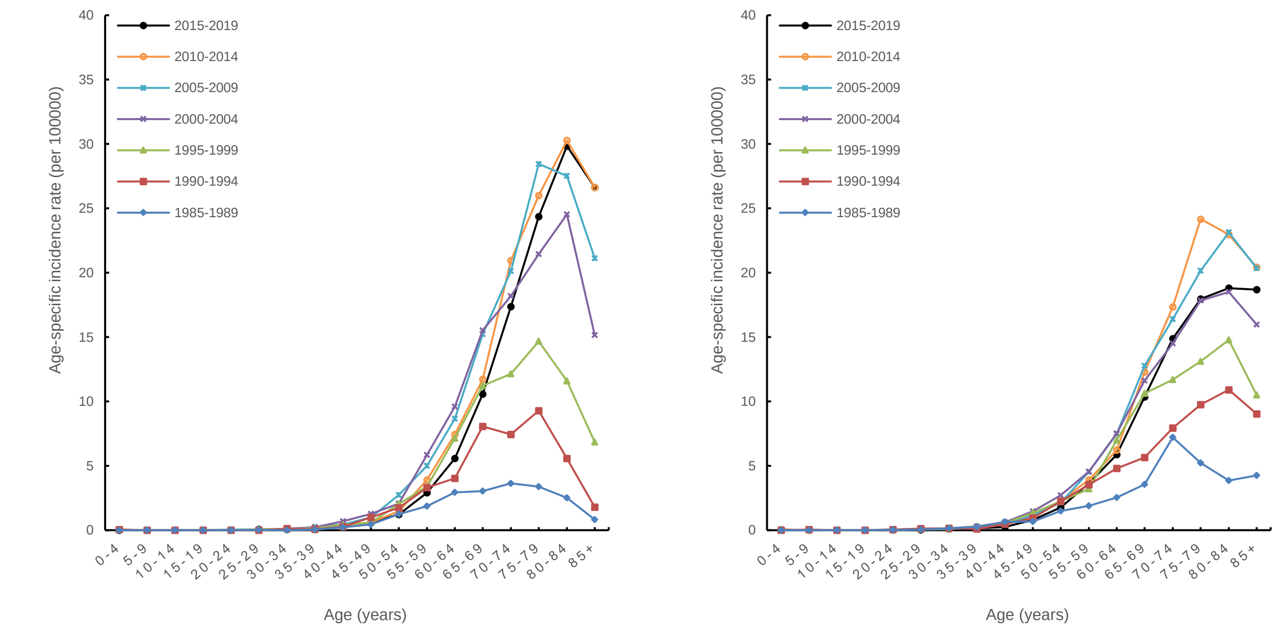

Supplementary Figure 1 Age-specific incidence rates for cancers of other urinary organs (A) in women and (B) in men.

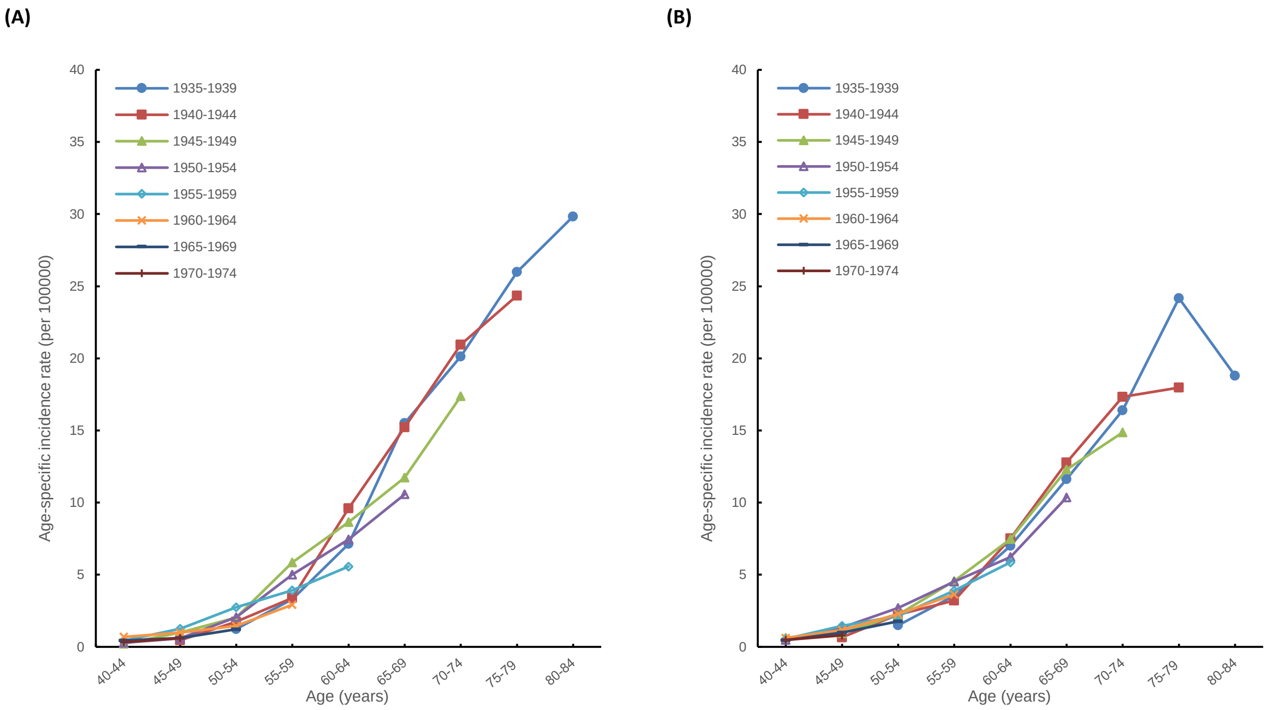

Supplementary Figure 2 Age-specific incidence rates by birth-year cohorts between 1935 and 1974 for cancers of other urinary organs (A) in women and (B) in men.
